# NGS-based biodiversity and community structure analysis of meiofaunal eukaryotes in shell sand from Hållö island, Smögen, and soft mud from Gullmarn Fjord, Sweden

**DOI:** 10.3897/BDJ.5.e12731

**Published:** 2017-06-08

**Authors:** Quiterie Haenel, Oleksandr Holovachov, Ulf Jondelius, Per Sundberg, Sarah J. Bourlat

**Affiliations:** 1 Zoological Institute, University of Basel, Basel, Switzerland; 2 Swedish Museum of Natural History, Stockholm, Sweden; 3 SeAnalytics AB, Bohus-Björkö, Sweden; 4 Department of Marine Sciences, University of Gothenburg, Gothenburg, Sweden

**Keywords:** Meiofaunal biodiversity, community structure, Illumina Mi-Seq, Metabarcoding, COI, 18S

## Introduction

Microscopic interstitial marine organisms, also termed ‘meiofauna’, are often defined as animals that pass a 1mm mesh but are retained on a 45 µm sieve (Higgins 1988). Meiofauna are an important component of sedimentary and benthic habitats due to their small size, abundance and rapid turnover rates. Moreover, meiofaunal surveys represent a useful tool for environmental impact assessments, underlying the urgent need for reliable, reproducible and rapid analytical methods. The breadth of taxonomic groups present in marine sediments makes meiofauna an ideal tool for detecting the effects of ecological impacts on marine biodiversity (Moreno et al. 2008). However, traditional morphology based taxonomy assignment methods are labour intensive and time consuming, leading us to explore recently developed metabarcoding methods for whole community analysis. Metabarcoding has previously been used to characterize plankton assemblages (Lindeque et al. 2013, de Vargas et al. 2015), marine benthic meiofaunal assemblages (Creer et al. 2010, Fonseca et al. 2014, Fonseca et al. 2010, Brannock and Halanych 2015, Cowart et al. 2015), meiofaunal communities colonizing autonomous reef monitoring structures (Leray and Knowlton 2015) or fish gut contents (Leray et al. 2013). The vast majority of studies have employed Roche 454 due to its long read lengths compared to other technologies (Table 1; Shokralla et al. 2012), but Illumina MiSeq is now able to provide similarly long reads using paired-end****sequencing (2x300 base pairs). As summarized in Table 1, there is no standardized method for metabarcoding of marine fauna, and a variety of sample extraction methods, sequencing platforms, molecular markers, bioinformatics pipelines and OTU clustering thresholds have been used to date, making these studies difficult to compare (Table 1).

In this study we used samples from muddy and sandy marine sediments to examine how results of metabarcoding based surveys of meiofaunal communities are impacted by three different meiofaunal extraction methods and three different primer pairs for COI and 18S. In order to validate the reliability of the metabarcoding approach, we compare the results obtained with traditional morphology-based taxonomic assignment for two test groups, Xenacoelomorpha and Nematoda, the latter previously shown to be the dominant taxon in meiofaunal communities in terms of number of OTUs (Fonseca et al. 2010).

## Materials and Methods

### Sampling

Samples were collected in two ecologically distinct locations along the west coast of Sweden in August 2014.

**Hållö island samples**: Coarse shell sand was sampled by dredging at 7-8m depth along the north-eastern side of Hållö island near Smögen, Sotenäs municipality, Västra Götalands county (N 58° 20.32-20.38', E 11° 12.73-12.68').

**Gullmarn Fjord samples**: Soft mud was collected using a Waren dredge at 53 m depth in the Gullmarn Fjord near Lysekil, Lysekil municipality, Västra Götalands county (N 58°15.73', E 11°26.10').

### Meiofaunal extraction

**Hållö island. **Hållö island samples were extracted in the lab using two different variations of the flotation (decanting and sieving) technique.

**Flotation (freshwater)**: Freshwater was used to induce an osmotic shock in meiofaunal organisms and force them to detach from heavy sediment particles. 200 mL of sediment were placed in a large volume of fresh water and thoroughly mixed to suspend meiofauna and lighter sediment particles. The supernatant was sieved through a 1000 µm sieve to separate the macrofaunal fraction, which was then discarded. The filtered sample was sieved again through a 45 µm sieve to collect meiofauna and discard fine organic particles. This procedure was repeated three times. Meiofauna was then rinsed with seawater from the sieve into large falcon tubes. Twelve sediment samples were processed, ten of them were fixed immediately in 96% ethanol for molecular analysis and stored at -20°C. The other two samples were first screened for live representatives of Xenacoelomorpha, and later preserved in 4% formaldehyde for morphology-based identification of nematodes.

**Flotation (MgCl2 solution)**: A 7.2% solution of MgCl2 was used to anesthetize meiofauna. As above, twelve samples were processed in total, ten of them were decanted through 125 µm sieve and fixed immediately in 96% ethanol for molecular analysis and stored at -20°C, while two samples were decanted through a 125 µm sieve which was subsequently placed in a petri dish with seawater. After 30 minutes, the petri dish as well as the inside of the sieve were searched for Xenacoelomorpha using a stereo microscope. Afterwards they were preserved in 4% formaldehyde for morphology-based identification of nematodes.

**Gullmarn Fjord. **Meiofauna was extracted from the Gullmarn Fjord samples using two different methods: flotation and siphoning.

**Flotation (freshwater)**: Freshwater was used to induce an osmotic shock in meiofaunal organisms. 2.4 L of sediment were placed in a large volume of freshwater, thoroughly mixed to suspend meiofauna and lighter sediment particles. The supernatant was sieved through a 1000 µm sieve in order to separate macrofauna, which was then discarded. The filtered sample was then sieved three times through a 70µm sieve to collect meiofauna and discard fine organic particles. Meiofauna was then rinsed with seawater from the sieve into a large container and equally divided between 12 falcon tubes. Six samples were fixed in 96% ethanol for molecular analysis and stored at -20°C. Six samples were screened for live representatives of Xenacoelomorpha, and preserved in 4% formaldehyde for morphology-based identification of nematodes.

**Siphoning: **A total volume of 12 L of sediment was processed as follows: an approximately 5 cm thick layer of mud was placed in a container and covered with 20 cm of seawater.  The sediment was allowed to settle for 20 hours. Half of the sediment area was then siphoned through a 125 µm sieve, the residue in the sieve was immediately fixed in 96% ethanol, large macrofauna was manually removed, and the entire volume was split equally into six samples and placed at -20°C for subsequent molecular analysis. The remaining half of the area was similarly siphoned through a 125 µm sieve, the sieve contents were stored in sea water, large macrofauna manually removed, the entire volume split into six samples, which were screened for live representatives of Xenacoelomorpha, and preserved in 4% formaldehyde for morphology-based identification of nematodes.

### Morphology-based identification

**Xenacoelomorpha**. Four samples from Hållö and 12 samples from Gullmarn Fjord were used for morphology-based assessment of the diversity of Xenacoelomorpha. All samples were stored in seawater and searched for Xenacoelomorpha with a stereo microscope. All specimens found were immediately identified to the lowest taxonomic rank possible using a compound microscope equipped with DIC.

**Nematoda**. Two samples from each location/extraction method were used to assess nematode diversity using morphology-based identification. Samples from Hållö (flotation with fresh water and MgCl2) and Gullmarn Fjord (siphoning) were processed whole and samples from Gullmarn Fjord extracted using flotation with fresh water were subsampled by taking 1/10 of the entire sample. Formaldehyde–preserved samples were transferred to glycerin using Seinhorst’s rapid method as modified by De Grisse (1969). Permanent nematode mounts on glass slides were prepared using the paraffin wax ring method. It is common practice to estimate the diversity of marine nematodes by counting a predetermined number (usually 100 or 200) of randomly picked nematodes per sample (Vincx 1996), which may not provide sufficiently detailed results for samples with high diversity. Therefore, all nematode specimens were counted and identified for each analyzed sample. All nematode specimens were identified to genus, and, when possible, to species level.

### DNA extraction, library preparation and sequencing

**DNA extraction. **30 samples were processed for total DNA extraction, twelve from the Gullmarn Fjord and eighteen from Hållö island, using 10g of sediment and the PowerMax^®^ Soil DNA Isolation Kit (MO BIO Laboratories), according to manufacturer’s instructions.

**Primer design.** Illumina MiSeq reagent v3. produces paired-end reads of 300bp in length, allowing a maximum marker length of 500bp when taking into account a 50 bp overlap. Universal COI primers available for the Metazoa amplify a 658bp region (Folmer et al. 1994), which is too long for most NGS applications.

Accordingly, primers amplifiying a 313 bp fragment of the mitochondrial cytochrome oxidase 1 (COI) gene were used, as described in Bourlat et al. 2016. The primers used for COI are modified from Leray et al.’s ‘mini-barcode’ COI primers (mlCOIintF-dgHCO2198; Leray et al. 2013) by adding the Illumina MiSeq overhang adapter sequences. The Leray et al. ‘mini-barcode’ primers have been shown to amplify up to 91% of metazoan diversity in a sample (Leray et al. 2013). In combination with Leray et al.'s mini barcode forward primer (mlCOIintF), we used Folmer et al.'s COI reverse primer (dgHCO2198; Folmer et al. 1994) as well as a reverse primer developed by Lobo et al., shown to enhance amplification of the COI region in a wide range of invertebrates (Lobo et al. 2013).

For the 18S region, Illumina overhang adapter sequences were appended to the primers from Fonseca et al. (SSU_FO4-SSU_R22; Fonseca et al. 2010), yielding a 364 bp fragment. These primers target a homologous region of the gene and flank a region that is highly divergent, corresponding to the V1-V2 region of the 18S gene (Lindeque et al. 2013, Fonseca et al. 2010).

Sequence overlap in the paired-end reads was calculated in Geneious Kearse et al. 2012. COI shows a sequence overlap of 230 bp and 18S shows an overlap of 190 bp.

All primer sequences used are shown in Table 2.

**Illumina MiSeq library preparation using fusion primers. **For Illumina MiSeq library preparation, we used a dual PCR amplification method as described in*Bourlat et al. (2016)*. The first PCR, the amplicon PCR, uses amplicon specific primers including the Illumina adapter overhang, as described above. The second PCR, the index PCR, allows the incorporation of Illumina index adapters using a limited number of cycles (Bourlat et al. 2016).

**Amplicon PCR. **PCR amplifications of the COI and 18S regions were set up as follows. For a 50µl reaction volume, we used 5µl Pfu polymerase buffer (10x), 1µl dNTP mix (final concentration of each dNTP 200µM), 0.5 µl of each primer at 50 pm/µl, 2 µl DNA template (~10 ng), 0.5µl Pfu DNA polymerase (Promega) and 40.5µl of nuclease free water. Each DNA sample was amplified with the 3 primer pairs described above (COI Leray, COI Lobo and 18S). PCR cycling conditions were 2 min at 95°C (1 cycle); 1 min at 95°C, 45 s at 57°C, 2 min at 72°C (35 cycles); 10 min at 72°C (1 cycle). The PCR was checked on a 2% agarose gel. 20µl of each PCR reaction were then purified with Agencourt^®^ AMPure^®^ XP paramagnetic beads (Beckman Coulter), allowing size selection of PCR fragments by using different PCR product to bead ratios (Bourlat et al. 2016).

**Index PCR. **For dual indexing we used the Nextera XT index kit (96 indices, 384 samples, Illumina) according manufacturers’ instructions. Dual indexing allows an increase in the multiplex level of sequencing per lane, so that more samples can be sequenced on the same flow cell (Fadrosh et al. 2014). It also eliminates cross-contamination between samples and the occurrence of mixed clusters on the flow cell (Kircher et al. 2012). The index PCR was set up as 50µl reactions using 5µl of cleaned up PCR amplicons, 5µl of Nextera XT Index Primer i5, 5µl of Nextera XT Index Primer i7, 25µl of 2x KAPA HiFi HotStart ready mix (Kapa Biosystems) and 10µl of nuclease free water. PCR cycling conditions were: 3 min at 95°C (1 cycle); 30 s at 95°C, 30 s at 55°C, 30 s at 72°C (8 cycles); 5 min at 72°C (1 cycle). A bead purification was carried out after the index PCR with Agencourt^®^ AMPure^®^ XP magnetic beads (Beckman Coulter) using a ratio of 0.8, allowing the selection of fragments larger than 200 bp. DNA was quantified before sequencing using a Qubit Fluoremeter (Invitrogen) and average fragment size was verified using Tapestation (Agilent Technologies). Further library normalization and pooling steps are described in*Bourlat et al. (2016)*.

**Sequencing. **The pooled libraries were sequenced three times independently using Illumina MiSeq Reagent Kit v3, producing in total 24 132 875 paired-end reads of 300 bp in length, of which 15 883 274 COI reads and 8 249 601 18S reads (Table 3).

### Bioinformatic data processing and analysis

**Figure 1.**
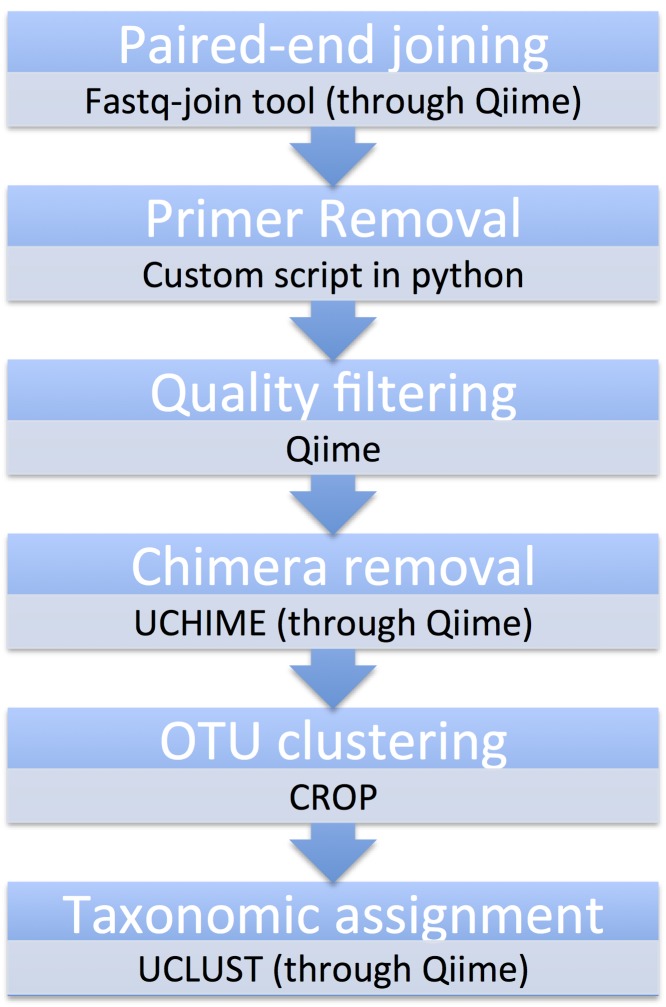
Schematic workflow of bioinformatic analytical steps

Most analytical steps were performed using Qiime (Quantitative Insight Into Microbial Ecology) version 1.9.1 (Caporaso et al. 2010) and custom python scripts (Fig. 1).

### Data resources

The data underpinning the analysis reported in this paper are deposited at the GenBank SRA under project number PRJNA388326 (https://www.ncbi.nlm.nih.gov/bioproject/PRJNA388326).

## Results and discussion

### Phylum-level community composition of meiofaunal samples from the Swedish west coast

**Figure 2.**
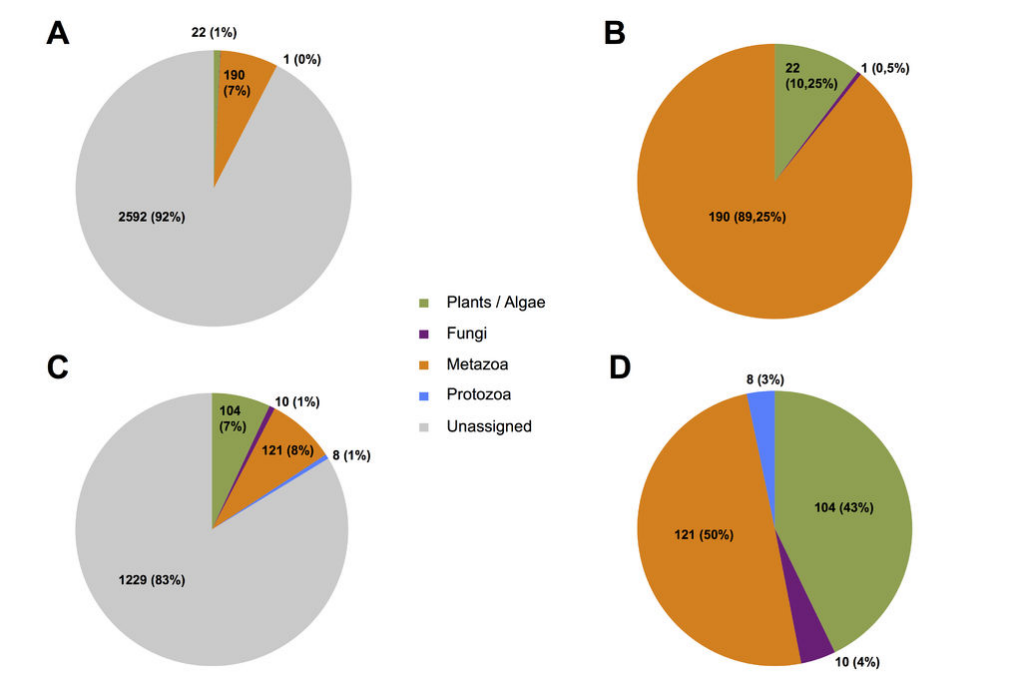
Taxonomic composition overview at species level based on a 97% sequence similarity threshold. A) Percentages and counts of OTUs for the COI gene with unassigned OTUs. B) Percentages and counts of OTUs for the COI gene without unassigned OTUs. C) Percentages and counts of OTUs for the 18S gene with unassigned OTUs. D) Percentages and counts of OTUs for the 18S gene without unassigned OTUs.

Illumina MiSeq produced at total of 24 132 875 raw reads, of which 15 883 274 COI reads and 8 249 601 18S reads. These were quality filtered (see methods section for details) resulting in 7 954 017 COI sequences and 890 370 18S sequences. These were clustered into 2805 and 1472 representative OTUs respectively, yielding 190 metazoan OTUs for COI and 121 metazoan OTUs for 18S at 97% sequence similarity (see methods, Table 5 & Fig. 2).

Taxonomic assignment of OTUs at a 97% similarity threshold shows community composition of the samples at the phylum level (Fig. 2). Of 2805 COI OTUs, 190 (7%) were assigned to the Metazoa, 22 (1%) to plants and algae, 1 (0%) to Fungi. 2592 OTUs remained unassigned, corresponding to 92% of COI OTUs.

For the 18S dataset, 121 of 1472 OTUs (8%) were assigned to Metazoa, 104 (7%) to plants and algae, 10 (1%) to Fungi, and 8 (1%) to Protozoa. 1229 OTUs remained unassigned, corresponding to 83% of all 18S OTUs.

The large numbers of unassigned OTUs reflect the incompleteness of the databases used for COI and 18S. When unassigned OTUs are disregarded, differences between the taxonomic ocverage of the markers can be observed (Fig. 2, B and D). COI is the ‘standard’ animal barcode and is thus mostly useful for diversity surveys within the Metazoa (Hebert et al. 2003). 18S has on the other hand much larger taxonomic coverage and can be used for biodiversity profiles of whole eukaryotic communities, at higher taxonomic scales.

**Figure 3.**
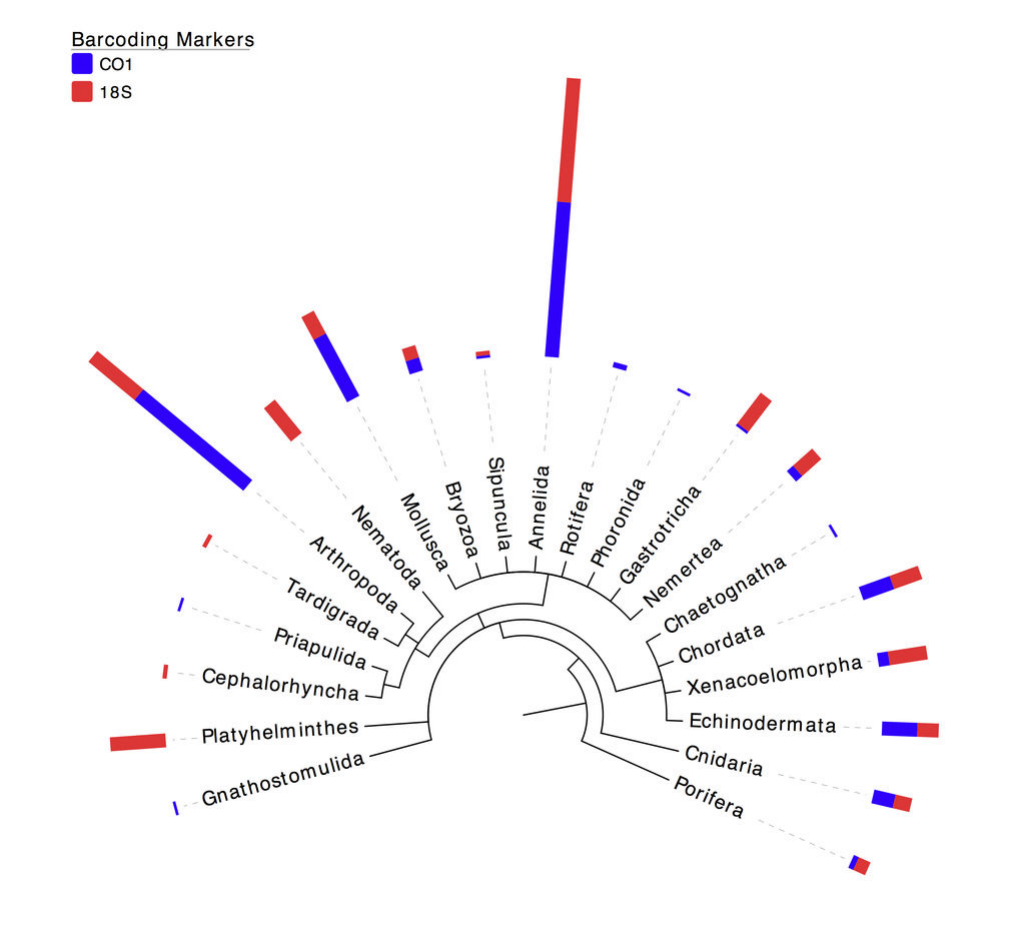
Percentages of metazoan phyla uncovered in the samples using COI and 18S molecular surveys. Blue bars correspond to the cumulated frequencies of OTUs assigned to a specific phylum using the COI gene and red bars correspond to the cumulated frequencies of OTUs assigned to a specific phylum using the 18S gene. Taxonomic assignment is based on a 97% sequence similarity threshold.

Of all OTUs classified as Metazoa, a detailed breakdown per phylum is presented in Table 5 and Fig. 3. Annelida (30% of CO1 metazoan OTUs and 23.97% of 18S metazoan OTUs) and Arthropoda (27.37% of CO1 metazoan OTUs and 11.57% of 18S metazoan OTUs), were the most OTU rich phyla identified in all samples combined, a similar pattern as observed in a recent study on coastal seagrass meadows in Brittany, France (Cowart et al. 2015).

As well as Annelida and Arthropoda, other phyla represented by a high number of OTUs in our samples include Mollusca (13.68% of COI metazoan OTUs and 4.96% of 18S metazoan OTUs), Platyhelminthes (10,74% of 18S metazoan OTUs and 0% of CO1 metazoan OTUs) and Nematoda (8.26% of 18S metazoan OTUs and 0% of CO1 metazoan OTUs) (Table 5 & Fig. 3). Other benthic metabarcoding studies based on the 18S V1-V2 region, found Nematoda and Platyhelminthes as the most OTU rich phyla represented (Fonseca et al. 2014, Fonseca et al. 2010), or Nematoda and Annelida (Bik et al. 2012b), alternatively Nematoda and Arthropoda (Bik et al. 2012a, Lallias et al. 2015).

### Meiofaunal community composition differs according to location

**Figure 4.**
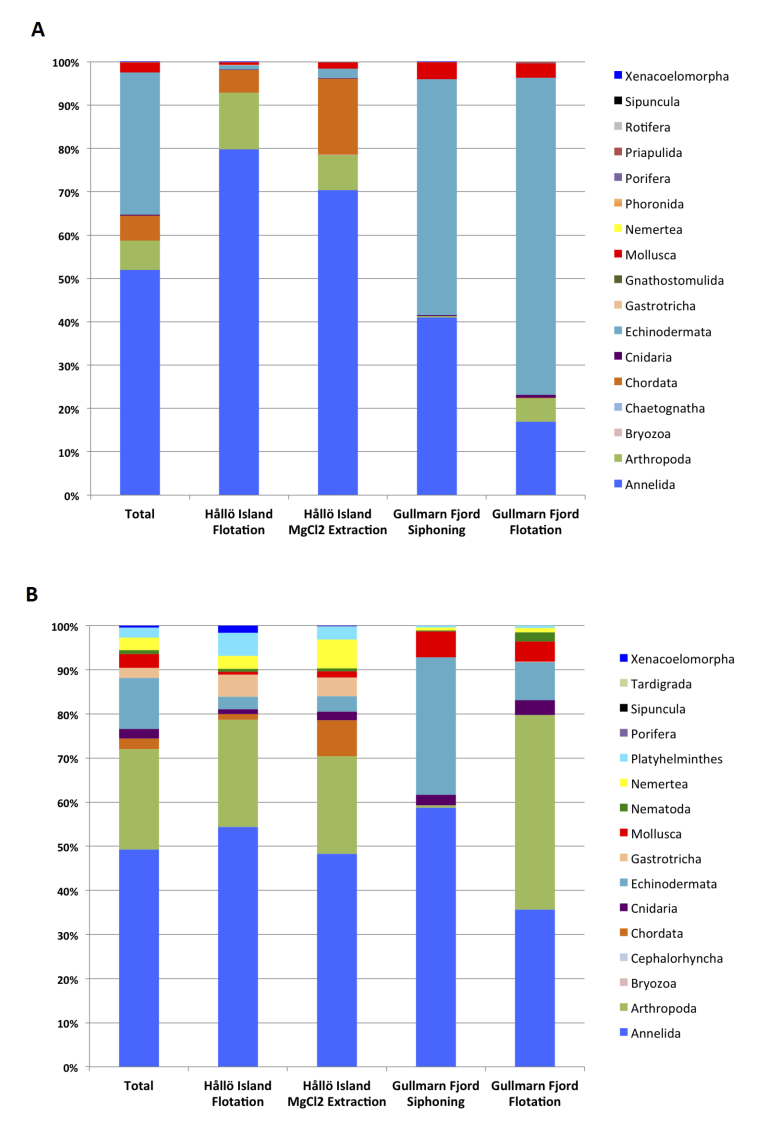
Community composition per phylum in Hållö island and Gullmarn fjord samples, according to extraction method (MgCl2, H2O, Siphoning). A) For the COI gene. B) For the 18S gene. The vertical axis corresponds to percentage of OTUs. Taxonomic assignment is based on a 97% similarity threshold. The bar plots take into account number of reads for each OTU.

Taxonomic community composition at both locations surveyed is illustrated in Fig. 4. The bar plots in Fig. 4 take into account the read counts for each OTU, whereas Table 5 and Fig. 3 do not take these into account.

In Fig. 4, clear differentiation in biodiversity between the two habitat types (soft mud versus coarse shell sand) can be observed, as expected. Echinodermata (such as Ophiurida, Echinoidea and Asteroidea), Mollusca (Bivalvia, Gastropoda), Annelida and Arthropoda are represented by higher numbers of reads in samples from the muddy sediments in the Gullmarn fjord samples (grain size 100 μm approx.).

In coarse shell sand in shallow areas, such as in the Hållö island samples, Annelida and Arthropoda are represented by higher numbers of reads, followed by Chordata (cephalohordata such as *Branchiostoma* sp., ascidians and various fish species such as *Gobius* sp., *Ctenolabrus rupestris, Solea solea*) with in addition a larger diversity of small taxa such as Bryozoa, Gnathosthomulida, Gastrotricha, Tardigrada, Rotifera, Sipuncula and Phoronida, reflecting the high diversity of insterstitial taxa found in sandy sediments.

### Sample diversity and composition analyses

**Figure 5.**
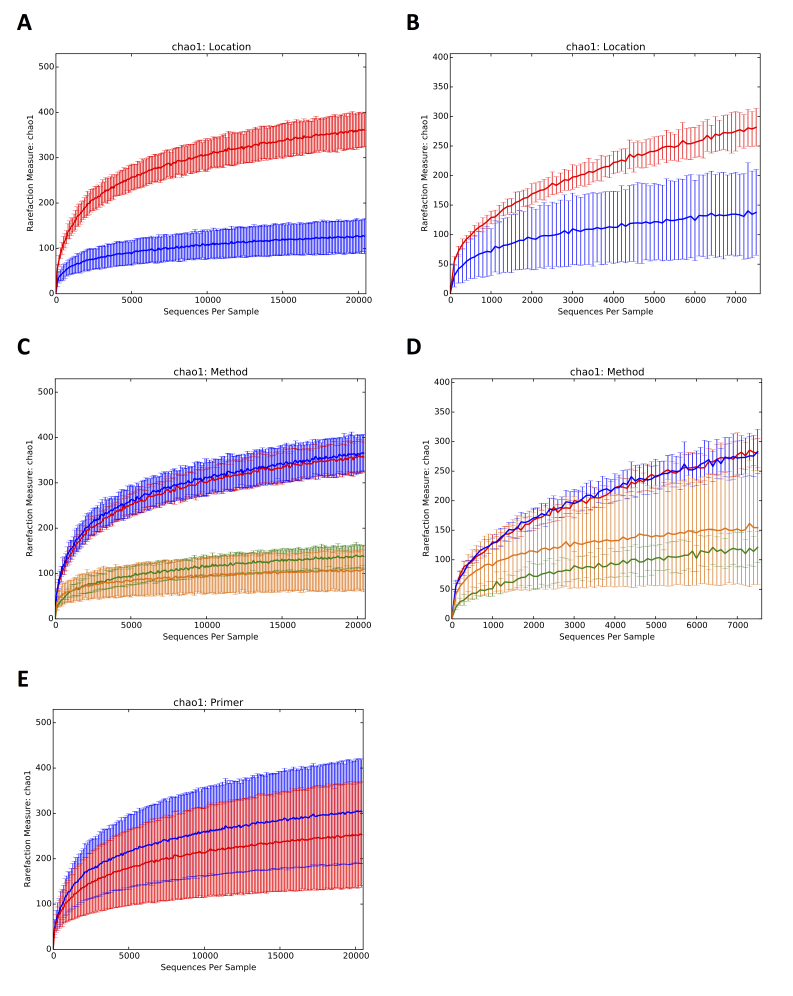
Alpha diversity rarefaction plots for COI and 18S datasets including unassigned OTUs. According to location for COI (A) 18S (B). Hållö Island (HI) in red, Gullmarn Fjord (GF) in blue. According to extraction method for COI (C) 18S (D). HI flotation in red, HI MgCl2 in blue, GF flotation in yellow, GF siphoning in green. According to primer pair for COI (E). CO1 Leray primer in red, COI Lobo primer in blue.

**Figure 6.**
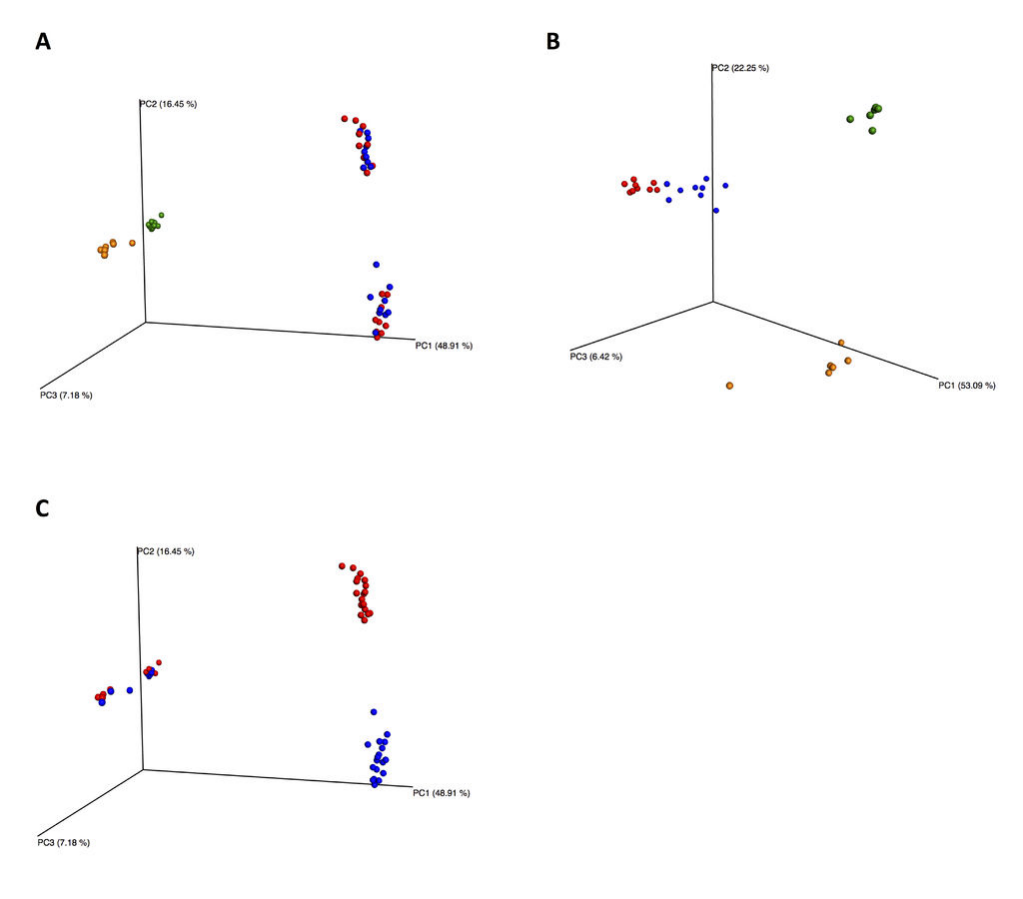
Beta diversity PCoA plots for COI and 18S datasets including unassigned OTUs. According to extraction method for COI (A) 18S (B) HI flotation in red, HI MgCl2 in blue, GF flotation in yellow and GF siphoning in green. According to primer for COI (C) COI Leray primer in red, COI Lobo primer in blue

A greater number of phyla were uncovered in the Hållö Island samples than in the Gullmarn Fjord samples (Fig. 4A and 4B) and this observation was corroborated by the alpha diversity rarefaction plots showing that Hållö Island samples (in red) present a higher diversity than the Gullmarn Fjord samples (in blue) (p-value = 0.001) regardless of the marker used (Fig. 5A and 5B). Within the same location, choice of extraction method does not have a significant impact on sample diversity (p-value ~ 1) (Fig. 5C and 5D, Table 6). However, for the 18S dataset, the flotation method seems to be more effective for extraction of nematodes than the siphoning method in the Gullmarn Fjord samples (Fig. 4A and 4B). Moreover, the beta diversity PCoA results highlight the fact that sample composition is influenced by the choice of extraction method for both COI and 18S datasets (p-value = 0.001) leading to four different clusters (Fig. 6and 6B, Table 6). For the COI dataset, in addition to extraction method as a factor of divergence, choice of primer (COI Leray or COI Lobo) also influences the grouping of the samples (p-value = 0.003 excluding unassigned OTUs and 0.001 including unassigned OTUs), in particular for the Hållö Island samples (Fig. 6C). Moreover, the COI Lobo primer seems to uncover a higher diversity of taxa than the COI Leray primer Fig. 5E) even if the results are considered to be non significant (p-value = 0.585 excluding unassigned OTUs and 0.111 including unassigned OTUs) (Table 6Table 7).

### Molecular identifications to species level

Using a sequence similarity search at 97% similarity allowed us to identify 213 COI OTUs and 243 18S OTUs to species level (Table 8 and Suppl. material 1). For the COI dataset, 81 species (of which 70 metazoans) were found in both locations, 36 (of which 35  metazoans) were found in the Gullmarn fjord only and 96 (of which 85 metazoans) were found in Hållö island only. For the 18S dataset, 108 species (of which 48 metazoans) were found in both locations, 44 (of which 21 metazoans) were found in the Gullmarn fjord only and 91 (of which 52 metazoans) were found in Hållö Island only (Suppl. material 1). These species observations from metabarcoding represent 'molecular occurrence records' that could be used in monitoring and other types of biodiversity surveys, in the same way as physical observations, such as for mapping species distributions (Bohmann et al. 2014, Lawson Handley 2015).

### Invasive and alien species detected in the samples

Five alien species were detected in in the sample, of which two are considered invasive (in bold; Table 9), and the other three are on alert lists. The two invasive species (*Acartia tonsa*, a copepod, and *Alexandrium ostenfefeldii*, a dinoflagellate) could easily be overlooked in routine monitoring programs. Species within the genus Acartia are difficult to distinguish (Jensen 2010) and the invasive species can be confused with other native species. Also * A. ostenfeldii* is easily misidentified as other *Alexandrium* species; detailed thecal plate observation is often necessary for proper identification (Balech 1995).  This shows the potential of molecular techniques for monitoring  invasive species, and points to problems using traditional identification techniques. Many invasive species arrive in an area as spores, larvae or juveniles - all life stages that may be easily overlooked and problematic to identify to species level. Target barcoding of environmental DNA (eDNA) shows a great promise for detecting species without the need of costly sampling schemes. This would also allow for more random sampling in an area, increasing the probability of actually finding a species even when they occur in low numbers.

### Comparison of metabarcoding versus morphology-based identification of Xenacoelomorpha

Comparison of morphology-based assessment of Xenacoelomorpha diversity with metabarcoding using taxonomic assignments to the phylum level (with 80% similarity threshold; Suppl. materials 2, 3), shows that extraction procedures have strong impact on the effectiveness of morphology-based identification (Tables 10, 11). Using freshwater for extraction of Xenacoelomorpha rendered most of them unrecognizable and unidentifiable, but left their DNA intact and suitable for metabarcoding. No identifiable Xenacoelomorpha were found in the Hållö samples extracted using flotation with fresh water, while all specimens found in Gullmarn Fjord were treated together as one taxon "*Acoela* sp." for the lack of better alternative. Metabarcoding, on the other hand, recovered between 6 and 15 taxa (OTUs) from the Hållö samples  extracted using flotation with fresh water (Table 11), and up to 13 taxa (OTUs) from the same type of samples from the Gullmarn Fjord site (Table 11), depending on the barcoding region used. Just like for nematodes (see below), 18S barcodes always gave higher overall estimates of diversity (number of OTUs) compared to COI (Table 11). 18S also gave higher diversity estimates, compared to morphology-based identification for the Hållö samples extracted using flotation with MgCl2 (11 versus 7), but lower for the Gullmarn Fjord site samples extracted using siphoning (9 versus 15). COI Leray primers were less effective compared to the COI Lobo primers that recovered 2-6 OTUs more in all samples (Table 11). The most numerous of the morphologically identified species, *Mecynostomum tenuissimum*, was present with 120 specimens in the manually sorted samples, but was not detected at all in the 18S samples. Note that the 18S and COI sequences for all of the species identified in the visually sorted samples are present in the reference database. This raises the question of the efficiacy of using the SSU_FO4-SSU_R22 18 S fragment for metabarcoding of acoelomorphs. A recent study found a number of unknown xenacoelomorph taxa while data mining metabarcoding sequences from surveys of pelagial and deep benthic habitats (Arroyo et al. 2016). Unknown xenacoelomorph species may exist also at the moderate sampling depths we sampled in the Gullmarn Fjord. Our siphoning technique relies on migration of specimens to the sediment surface in response to hypoxia. It is possible that there are xenacoelomorphs with high tolerance for hypoxia that are not captured by the siphoning method, and thus would not be found in the manually sorted samples, but could be detected by metabarcoding of unprocessed samples. It should be noted that the extraction method used on the Hållö samples does not rely on migration of specimens to the surface.

### Comparison of metabarcoding versus morphology-based identification of Nematoda

Both study sites are characterized by rich and diverse nematode fauna. The Hållö site had a total of 107 species of nematodes, belonging to 86 genera (Holovachov et al. 2017). Of these, 88 species belonging to 73 genera were found in samples extracted by flotation with a MgCl2 solution, and 101 species belonging to 83 genera were found in samples extracted by flotation with fresh water. The Gullmarn fjord site had a total of 113 nematode species of nematodes, belonging to 77 genera (Holovachov et al. 2017). Of these, 81 species belonging to 62 genera were found in samples extracted by siphoning, and 102 species belonging to 70 genera were found in samples extracted by flotation with fresh water. A certain small number of nematode individuals in each sample were not identified to species/genus/family, either due to their developmental stage or quality of preservation.

The final list of nematode OTUs includes 139 18S sequences. Only two 18S OTUs were positively identified using QIIME to species level using 97% similarity threshold: *Viscosia viscosa* (TS6.SSU58722) and *Chromadora nudicapitata* (HF2.SSU192072), six more were assigned to reference sequences identified to genus level only (Suppl. material 1). Only 22 COI sequences were assigned to the phylum Nematoda, and none was identified to species level.

When comparing the results of morphology-based assessment of nematode diversity with metabarcoding using taxonomic assignments to the phylum level in this particular study (with 80% similarity threshold; Suppl. materials 2, 3), the detailed and extensive examination of samples and morphology-based species identification provided more comprehensive estimates of nematode diversity (107 species in Hållö and 113 species in Gullmarn Fjord) than metabarcoding using either one of the molecular markers, independently of the extraction technique or locality (Table 12). Moreover, COI barcodes were much harder to obtain for marine nematodes using either one of the primers (16 OTUs in Hållö and 9 OTUs in Gullmarn Fjord using Lobo primers; 17 OTUs in Hållö and 4 OTUs in Gullmarn Fjord using Leray primers), comparing to 18S (95 OTUs in Hållö and 78 OTUs in Gullmarn Fjord site; Table 12). Due to the very limited reference databases available for marine nematodes, very few nematode OTUs can be identified to species or genus level, making it difficult to use metabarcoding data in ecological studies.

 

## Supplementary Material

Supplementary material 1 OTUs identified to species level in the samples using 97% sequence similarity, all organism groups Data type: Occurrence records from Metabarcoding for Hållö island and Gullmarsfjord, Sweden.
Brief description: Sequence similarity search at 97% similarity allowed us to identify some OTUs to species level. 215 COI OTUs and 243 18S OTUs were identified to species from both sites (Hållö island and Gullmarsfjord).
File: oo_137797.xlsx Quiterie Haenel, Oleksandr Holovachov, Ulf Jondelius, Per Sundberg and Sarah J. Bourlat

Supplementary material 2 OTU table for 18S Data type: Metagenomic, OTU table
Brief description: OTU table showing all 18S OTUs, their taxonomic assignment at 80% similarity and number of reads per sample (HE: Hållö Flotation, HF: Hållö Flotation MgCl2, TS: Gullmarn Fjord Siphoning, TF: Gullmarn Fjord Flotation)
File: oo_124225.txt Quiterie Haenel, Oleksandr Holovachov, Ulf Jondelius, Per Sundberg and Sarah J. Bourlat

Supplementary material 3 OTU table for COI Data type: Metagenomic, OTU table
Brief description: OTU table showing all COI OTUs, their taxonomic assignment at 80% similarity and number of reads per sample (HE: Hållö Flotation, HF: Hållö Flotation MgCl2, TS: Gullmarn Fjord Siphoning, TF: Gullmarn Fjord Flotation)
File: oo_124226.txt Quiterie Haenel, Oleksandr Holovachov, Ulf Jondelius, Per Sundberg and Sarah J. Bourlat

## Figures and Tables

**Table 1.** Methodological comparison of benthic and pelagic metabarcoding studies of marine fauna published to date

**Authors**	**Sample type**	**Sample extraction method**	**Sequencing platform**	**Marker**	**Marker size (bp)**	**Chimera screening**	**OTU clustering method and threshold**	**Database**
Leray et al. 2013	Coral reef fish gut contents	Dissection of fish gut	Roche 454 GS FLX	COI	313	UCHIME	CROP 92-94%	Moorea Biocode Database, GenBank
Leray and Knowlton 2015	Autonomous reef monitoring structures	4 fractions (Sessile, 2mm, 500μm, 106μm)	Ion Torrent	COI	313			BOLD, GenBank
Lindeque et al. 2013	Zooplankton from 50m to the surface	200μm mesh WP2 plankton net	Roche 454 GS FLX	18S (V1-V2 regions)	450	ChimeraSlayer (QIIME 1.3.0)	UCLUST 97% (QIIME 1.3.0)	Silva 108, GenBank
de Vargas et al. 2015	Plankton	3 fractions (5-20μm, 20-180μm, 180-2000μm)	Paired-end Illumina Genome Analyser IIx system	18S (V9 region)		USEARCH		V9_PR2, V9 rDNA, Protistan Ribosomal Reference Database
Fonseca et al. 2010	Marine benthic meiofauna	Decanting 45μm sieve Ludox	Roche 454 GS FLX	18S (V1-V2 regions)	364 (250-500)	OCTOPUS	OCTOPUS 96%	GenBank
Fonseca et al. 2014	Marine benthic meiofauna	Decanting 45μm sieve Ludox	Roche 454 GS FLX	18S (V1-V2 regions)	450	Amplicon-Noise	Amplicon-Noise 99% and 96%	GenBank
Brannock and Halanych 2015	Marine benthic meiofauna	Directly from sediment, elutriated on 45μm sieve	Paired-end 100 bp reads Illumina HiSeq	18S (V9 region)	87-187 [13]	USEARCH 6.1. (QIIME 1.8)	UPARSE 97% UCLUST and USEARCH (QIIME 1.8)	Silva 111
Cowart et al. 2015	Benthic meiofauna from seagrass meadows	2mm sieve, 1mm sieve, 0.5mm sieve	Roche 454 GS FLX	COI 18S	450 710	USEARCH 6.1 (QIIIME 1.7)	UCLUST de novo (QIIME 1.7)	GenBank Silva 115
This study	Meiofauna from coarse shell sand and muddy benthic sediment	Siphoning 125μm, flotation (MgCl2) 125μm, flotation (H2O) 45μm/70μm	Paired-end Illumina Mi-Seq	COI 18S (V1-V2 regions)	313 364	UCHIME (part of USEARCH 6.1.) (QIIME 1.9.1)	CROP COI: 92-94% 18S: 95-97%	BOLD, SweBol and own databases for Nemertea, Acoela, Oligochaeta), Genbank Silva 111

**Table 2.** Primer sequences used in this study

**Marker**	**Primer name**	**Illumina adapter overhang (regular font), with primer sequence (in bold)**
**COI Leray**	mlCOIintF	5’-TCGTCGGCAGCGTCAGATGTGTATAAGAGACAG**GGWACWGGWTGAACW GTWTAYCCYCC**-3’
	dgHCO2198	5’-GTCTCGTGGGCTCGGAGATGTGTATAAGAGACAG**TAAACTTCAGGGTGAC CAAARAAYCA**-3’
**COI Lobo**	mlCOIintF	5’-TCGTCGGCAGCGTCAGATGTGTATAAGAGACAG**GGWACWGGWTGAACW GTWTAYCCYCC**-3’
	LoboR1	5’-GTCTCGTGGGCTCGGAGATGTGTATAAGAGACAG**TAAACYTCWGGRTGW CCRAARAAYCA**-3’
**18S**	SSU_FO4	5’-TCGTCGGCAGCGTCAGATGTGTATAAGAGACAG**GCTTGTCTCAAAGATTA AGCC**-3’
	SSU_R22	5’-GTCTCGTGGGCTCGGAGATGTGTATAAGAGACAG**GCCTGCTGCCTTCCTT GGA**-3’

**Table 3.** Number of reads per marker and per sequencing run

**Marker / Sequencing run**	**1**	**2**	**3**	**Total**
COI	5 859 454	5 075 735	4 948 085	15 883 274
18S	2 803 391	3 135 331	2 310 879	8 249 601
Total	8 662 845	8 211 066	7 258 964	24 132 875

**Table 4.** Number of reads remaining after each bioinformatic step

**Marker / Step**	**Raw data**	**Paired-end joining**	**Primer trimming**	**Quality filtering**	**Chimera removal**
COI	15 883 274	10 412 096	8 099 507	7 976 649	7 954 017
18S	8 249 601	2 131 102	1 071 871	1 015 874	890 370
Total	24 132 875	12 543 198	9 171 378	8 992 523	8 844 387

**Table 5.** Number of OTUs and percentage per phylum for COI and 18S for the metazoan fraction. Based on a 97% similarity threshold.

**Phylum**	**COI**		**18S**	
	**OTUs**	**Percentage**	**OTUs**	**Percentage**
Annelida	57	30.00	29	23.97
Arthropoda	52	27.37	14	11.57
Bryozoa	5	2.63	3	2.48
Cephalorhyncha	0	0.00	1	0.83
Chaetognatha	1	0.53	0	0.00
Chordata	12	6.32	7	5.79
Cnidaria	8	4.21	4	3.31
Echinodermata	13	6.84	5	4.13
Gastrotricha	1	0.53	9	7.44
Gnathostomulida	1	0.53	0	0.00
Mollusca	26	13.68	6	4.96
Nematoda	0	0.00	10	8.26
Nemertea	3	1.58	6	4.96
Platyhelminthes	0	0.00	13	10.74
Phoronida	1	0.53	0	0.00
Porifera	2	1.05	3	2.48
Priapulida	1	0.53	0	0.00
Rotifera	2	1.05	0	0.00
Sipuncula	1	0.53	1	0.83
Tardigrada	0	0.00	1	0.83
Xenacoelomorpha	4	2.11	9	7.44
Total OTUs Metazoa	190	100	121	100

**Table 6.** Nonparametric t-test results with 999 Monte-Carlo permutations for both datasets with and without unassigned OTUs (97% taxonomic assignment)

	**COI dataset**				**18S dataset**			
	Excluding unassigned OTUs		Including Unassigned OTUs		Excluding unassigned OTUs		Including Unassigned OTUs	
	Test value	P-value	Test value	P-value	Test value	P-value	Test value	P-value
Location								
HI vs. GF	-14.453	0.001	-21.455	0.001	-6.929	0.001	-7.170	0.001
Method								
HI H2O vs. HI MgCl2	-0.437	1.0	-0.691	1.0	-0.906	1.0	-0.174	1.0
GF flotation vs. GF siphoning	1.567	0.792	1.546	0.99	-1.427	1.0	-0.744	1.0
Primer								
COI Leray vs. COI Lobo	-0.508	0.596	-1.614	0.111	-	-	-	-

**Table 7.** ANOSIM test results (999 permutations) for both COI and 18S datasets with and without unassigned OTUs (97% taxonomic assignment)

	**COI dataset**				**18S dataset**			
Ho: Sample composition differs according to	Excluding unassigned OTUs		Including unassigned OTUs		Excluding unassigned OTUs		Including unassigned OTUs	
	R-value	P-value	R-value	P-value	R-value	P-value	R-value	P-value
Location	0.976	0.001	1.0	0.001	0.935	0.001	0.929	0.001
Method	0.660	0.001	0.738	0.001	0.889	0.001	0.895	0.001
Primer	0.200	0.003	0.218	0.001	-	-	-	-

**Table 8.** Metazoa identified to species level using 97% sequence similarity (HI: Hållö island, GF: Gullmarn Fjord)

**COI**							
OTU ID	Nb of reads	Phylum	Class	Order	Species	HI	GF
HE6.Lobo_7972794	3	Annelida	Clitellata	Haplotaxida	*Adelodrilus pusillus*	+	-
HE1.Lobo_933012	14954	Annelida	Clitellata	Haplotaxida	*Grania postclitellochaeta*	+	+
HF8.Lobo_5239705	241	Annelida	Clitellata	Haplotaxida	*Grania variochaeta*	+	+
HF4.Lobo_97092	29391	Annelida	Clitellata	Haplotaxida	*Tubificoides benedii*	+	+
HF5.Lobo_3297996	1	Annelida	Clitellata	Haplotaxida	*Tubificoides kozloffi*	+	-
TS1.Leray_545620	7370	Annelida	Polychaeta	Amphinomida	*Paramphinome jeffreysii*	-	+
HF1.Lobo_4996219	4596	Annelida	Polychaeta	Canalipalpata	*Polygordius appendiculatus*	+	+
TF6.Lobo_5247622	9030	Annelida	Polychaeta	Capitellida		-	+
TS1.Lobo_4669404	5	Annelida	Polychaeta	Capitellida		-	+
TF5.Lobo_6394093	2	Annelida	Polychaeta	Capitellida		-	+
TS3.Leray_6813257	1852	Annelida	Polychaeta	Eunicida		-	+
HF5.Leray_4035802	1	Annelida	Polychaeta	Eunicida	*Ophryotrocha maculata*	+	-
TS2.Leray_4445240	8815	Annelida	Polychaeta	Eunicida	*Parougia eliasoni*	+	+
TF3.Leray_6645504	5196	Annelida	Polychaeta	Opheliida		+	+
TS5.Lobo_6031643	5089	Annelida	Polychaeta	Opheliida		+	+
HF9.Lobo_7587930	1	Annelida	Polychaeta	Opheliida		+	-
HE8.Leray_7284535	2	Annelida	Polychaeta	Phyllodocida		+	-
TS5.Leray_1557252	88	Annelida	Polychaeta	Phyllodocida		-	+
TS3.Leray_6744085	1	Annelida	Polychaeta	Phyllodocida		-	+
TS3.Leray_6805306	2	Annelida	Polychaeta	Phyllodocida	*Aphrodita aculeata*	-	+
TS3.Lobo_1308935	4213	Annelida	Polychaeta	Phyllodocida	*Eumida ockelmanni*	+	+
HE6.Leray_2958692	69642	Annelida	Polychaeta	Phyllodocida	*Glycera alba*	+	+
HF7.Leray_1672792	69	Annelida	Polychaeta	Phyllodocida	*Glycinde nordmanni*	+	+
TF5.Leray_2872180	7754	Annelida	Polychaeta	Phyllodocida	*Gyptis mackiei*	-	+
HF1.Lobo_5059232	13	Annelida	Polychaeta	Phyllodocida	*Gyptis propinqua*	+	-
HF9.Lobo_7695035	1	Annelida	Polychaeta	Phyllodocida	*Lepidonotus squamatus*	+	-
HE6.Lobo_7972042	2	Annelida	Polychaeta	Phyllodocida	*Myrianida edwarsi*	+	-
HF9.Lobo_7688887	3	Annelida	Polychaeta	Phyllodocida	*Nereimyra punctata*	+	-
HF2.Lobo_2136301	178929	Annelida	Polychaeta	Phyllodocida	*Pisione remota*	+	+
HE3.Leray_364663	59407	Annelida	Polychaeta	Phyllodocida	*Platynereis dumerilli*	+	+
TS4.Leray_7471107	1	Annelida	Polychaeta	Phyllodocida	*Sige fusigera*	-	+
HE5.Lobo_493462	571790	Annelida	Polychaeta			+	+
TS2.Lobo_6962270	4595	Annelida	Polychaeta	Sabellida	*Galathowenia oculata*	+	+
TS2.Leray_4491798	316559	Annelida	Polychaeta	Spionida		+	+
TS4.Lobo_1502925	195999	Annelida	Polychaeta	Spionida		+	+
HF9.Lobo_7588557	891	Annelida	Polychaeta	Spionida		+	-
TS6.Leray_5665274	936	Annelida	Polychaeta	Spionida		-	+
TF1.Lobo_2668551	874	Annelida	Polychaeta	Spionida		-	+
HE4.Leray_3067470	3	Annelida	Polychaeta	Spionida	*Chaetopterus sarsi*	+	-
HF1.Lobo_4965916	1	Annelida	Polychaeta	Spionida	*Malacoceros fuliginosus*	+	-
HF9.Leray_4404528	1	Annelida	Polychaeta	Spionida	*Polydora cornuta*	+	-
HF5.Lobo_3178682	2894	Annelida	Polychaeta	Spionida	*Spiophanes bombyx*	+	+
TF1.Leray_2314881	29235	Annelida	Polychaeta	Terebellida		+	+
TF1.Lobo_2832834	9348	Annelida	Polychaeta	Terebellida		+	+
TS1.Leray_614419	788	Annelida	Polychaeta	Terebellida		+	+
HE8.Lobo_858951	1	Annelida	Polychaeta	Terebellida		+	-
TS2.Lobo_6889557	184	Annelida	Polychaeta	Terebellida		-	+
TS6.Lobo_255019	3	Annelida	Polychaeta	Terebellida		-	+
TS2.Lobo_6860909	1	Annelida	Polychaeta	Terebellida		-	+
TS5.Leray_1638640	1	Annelida	Polychaeta	Terebellida		-	+
TF1.Lobo_2848745	1305	Annelida	Polychaeta	Terebellida	*Amphictene auricoma*	+	+
TS3.Leray_6729893	1	Annelida	Polychaeta	Terebellida	*Brada villosa*	-	+
HF4.Lobo_96799	102	Annelida	Polychaeta	Terebellida	*Cirratulus cirratus*	+	-
HF2.Lobo_2052205	285	Annelida	Polychaeta	Terebellida	*Dodecaceria concharum*	+	-
TS5.Leray_1638834	102	Annelida	Polychaeta	Terebellida	*Lagis koreni*	+	+
HE9.Lobo_2191024	8	Annelida	Polychaeta	Terebellida	*Macrochaeta clavicornis*	+	+
TF1.Leray_2475372	6353	Annelida	Polychaeta	Terebellida	*Sosane wahrbergi*	+	+
HE1.Lobo_982378	38	Arthropoda	Branchiopoda	Diplostraca	*Evadne nordmanni*	+	-
TF5.Lobo_6391642	10097	Arthropoda	Branchiopoda	Diplostraca	*Penilia avirostris*	+	+
HF9.Lobo_7623741	1	Arthropoda	Branchiopoda	Diplostraca	*Pleopis polyphemoides*	+	-
TS4.Leray_7402581	10	Arthropoda	Insecta	Diptera		+	+
TS3.Lobo_1162454	2	Arthropoda	Insecta	Diptera	*Chironomus aprilinus*	+	+
HF4.Lobo_5006	1	Arthropoda	Insecta	Diptera	*Cryptochironomus supplicans*	+	-
TF5.Leray_2910679	6	Arthropoda	Insecta	Diptera	*Procladius* sp.	+	+
HF9.Lobo_7599310	3	Arthropoda	Insecta	Diptera	*Psectrocladius yunoquartus*	+	+
HE5.Lobo_479906	152	Arthropoda	Insecta	Diptera	*Tanytarsus usmaensis*	+	+
HE2.Lobo_2023271	21589	Arthropoda	Malacostraca	Amphipoda		+	+
HF1.Leray_2493444	3911	Arthropoda	Malacostraca	Amphipoda		+	-
HE8.Lobo_860608	1	Arthropoda	Malacostraca	Amphipoda		+	-
HE3.Lobo_4900763	1	Arthropoda	Malacostraca	Amphipoda	*Ampelisca brevicornis*	+	-
HF4.Leray_6193380	66039	Arthropoda	Malacostraca	Amphipoda	*Atylus vedlomensis*	+	+
HE8.Leray_7216397	1	Arthropoda	Malacostraca	Amphipoda	*Corophium volutator*	+	-
HE6.Lobo_7849183	1	Arthropoda	Malacostraca	Amphipoda	*Leptocheirus hirsutimanus*	+	-
HE1.Lobo_914374	14588	Arthropoda	Malacostraca	Amphipoda	*Monocorophium insidiosum*	+	+
TF1.Leray_2445583	56	Arthropoda	Malacostraca	Amphipoda	*Monoculodes packardi*	-	+
TF6.Leray_5321299	11588	Arthropoda	Malacostraca	Cumacea		+	+
HF9.Leray_4291607	1372	Arthropoda	Malacostraca	Decapoda	*Athanas nitescens*	+	-
HF8.Leray_5586003	2864	Arthropoda	Malacostraca	Decapoda	*Eualus cranchii*	+	+
HF8.Leray_5612792	37	Arthropoda	Malacostraca	Decapoda	*Eualus cranchii*	+	-
HE1.Lobo_952576	3739	Arthropoda	Malacostraca	Decapoda	*Liocarcinus navigator*	+	-
TF5.Lobo_6459477	1279	Arthropoda	Malacostraca	Decapoda	*Philocheras bispinosus bispinosus*	+	+
HE4.Lobo_4138563	42	Arthropoda	Malacostraca	Decapoda	*Pisidia longicornis*	+	+
HE8.Leray_7306131	2	Arthropoda	Malacostraca	Decapoda	*Processa modica*	+	-
TS3.Lobo_1213146	17	Arthropoda	Malacostraca	Isopoda	*Asellus aquaticus*	+	+
TF5.Leray_2897128	3	Arthropoda	Maxillopoda	Calanoida	*Acartia bifilosa*	-	+
HF3.Leray_7129076	22	Arthropoda	Maxillopoda	Calanoida	*Acartia clausi*	+	+
TF6.Leray_5332240	7399	Arthropoda	Maxillopoda	Calanoida	*Acartia tonsa*	+	+
HF7.Leray_1683272	927	Arthropoda	Maxillopoda	Calanoida	*Acartia tonsa*	+	+
HE2.Lobo_2010882	1	Arthropoda	Maxillopoda	Calanoida	*Anomalocera patersoni*	+	-
TS2.Leray_4478240	2	Arthropoda	Maxillopoda	Calanoida	*Calanus euxinus*	-	+
HF7.Lobo_5810493	41	Arthropoda	Maxillopoda	Calanoida	*Centropages hamatus*	+	+
HF8.Lobo_5106754	82	Arthropoda	Maxillopoda	Calanoida	*Centropages typicus*	+	+
HE8.Leray_7251655	1	Arthropoda	Maxillopoda	Calanoida	*Eurytemora affinis*	+	-
HE7.Leray_3803390	5325	Arthropoda	Maxillopoda	Calanoida	*Paracalanus parvus*	+	+
HF9.Leray_4411242	1	Arthropoda	Maxillopoda	Calanoida	*Pseudocalanus elongatus*	+	-
TS4.Leray_7515925	2	Arthropoda	Maxillopoda	Calanoida	*Pseudocalanus elongatus*	-	+
TS3.Lobo_1208165	1	Arthropoda	Maxillopoda	Calanoida	*Scolecithricella minor*	-	+
TF5.Lobo_6373065	809	Arthropoda	Maxillopoda	Calanoida	*Temora longicornis*	+	+
TF1.Leray_2453024	1	Arthropoda	Maxillopoda	Calanoida	*Temora longicornis*	-	+
HF4.Leray_6242499	45	Arthropoda	Maxillopoda	Cyclopoida		+	-
HF4.Leray_6206299	2	Arthropoda	Maxillopoda	Harpacticoida		+	-
HE8.Lobo_823478	108	Arthropoda	Maxillopoda	Harpacticoida	*Harpacticoida* sp.	+	-
TS3.Lobo_1208905	116	Arthropoda	Maxillopoda	Harpacticoida	*Harpacticus flexus*	+	+
HE1.Lobo_995710	1	Arthropoda	Maxillopoda	Harpacticoida	*Tachidius discipes*	+	-
HF4.Leray_6092514	1	Arthropoda	Maxillopoda	Poecilostomatoida		+	-
HF9.Leray_4391714	11307	Arthropoda	Maxillopoda	Sessilia	*Balanus balanus*	+	+
HF4.Leray_6295260	1079	Arthropoda	Maxillopoda	Sessilia	*Balanus balanus*	+	+
HF7.Leray_1785147	2	Arthropoda	Maxillopoda	Sessilia	*Verruca stroemia*	+	-
HE1.Leray_1117391	1	Arthropoda	Pycnogonida	Pantopoda	*Endeis spinosa*	+	-
HE9.Lobo_2173983	63	Bryozoa	Gymnolaemata	Cheilostomatida	*Escharella immersa*	+	-
HF7.Leray_1838377	98	Bryozoa	Gymnolaemata	Cheilostomatida	*Membranipora membranacea*	+	-
HE3.Lobo_4881810	541	Bryozoa	Gymnolaemata	Cheilostomatida	*Scrupocellaria scruposa*	+	-
HF6.Lobo_2617384	2	Bryozoa	Gymnolaemata	Ctenostomata	*Amathia gracilis*	+	-
HF5.Lobo_3158598	5	Bryozoa	Stenolaemata	Cyclostomatida	*Crisia eburnea*	+	-
HE6.Leray_2983148	31	Chaetognatha	Sagittoidea	Aphragmophora		+	-
TS1.Leray_646185	73	Chordata	Actinopterygii	Gasterosteiformes	*Gasterosteus aculeatus*	+	+
HF4.Lobo_208606	1	Chordata	Actinopterygii	Perciformes	*Ammodytes marinus*	+	-
HF1.Leray_2487062	288	Chordata	Actinopterygii	Perciformes	*Ctenolabrus rupestris*	+	-
HF3.Lobo_3538759	472	Chordata	Actinopterygii	Perciformes	*Gobius niger*	+	-
TF1.Lobo_2807051	486	Chordata	Actinopterygii	Perciformes	*Lesueurigobius friesii*	+	+
HF9.Lobo_7596943	8	Chordata	Actinopterygii	Perciformes	*Mullus surmuletus*	+	-
HF5.Lobo_3273051	43	Chordata	Actinopterygii	Perciformes	*Trachinus draco*	+	-
HE2.Lobo_1914646	81	Chordata	Actinopterygii	Pleuronectiformes	*Limanda limanda*	+	-
HE8.Lobo_879846	265	Chordata	Actinopterygii	Pleuronectiformes	*Solea solea*	+	-
HE8.Lobo_756051	34	Chordata	Actinopterygii	Salmoniformes	*Salmo trutta*	+	-
HF3.Lobo_3595218	14	Chordata	Ascidiacea	Phlebobranchia	*Phallusia ingeria*	+	-
HE8.Lobo_873511	131011	Chordata	Leptocardii	-	*Branchiostoma lanceolatum*	+	+
TF3.Leray_6588680	3869	Cnidaria	Anthozoa	Pennatulacea	*Funiculina* sp.	+	+
TF6.Lobo_5251371	1	Cnidaria	Hydrozoa	Anthoathecata	*Corymorpha nutans*	-	+
HE9.Lobo_2164485	2	Cnidaria	Hydrozoa	Anthoathecata	*Lizzia blondina*	+	-
TF6.Leray_5512978	1481	Cnidaria	Hydrozoa	Leptothecata	*Eutima gracilis*	+	+
HF5.Lobo_3253786	232	Cnidaria	Scyphozoa	Semaeostomeae	*Aurelia aurita*	+	+
HE3.Leray_361248	14	Cnidaria	Scyphozoa	Semaeostomeae	*Cyanea capillata*	+	+
HE2.Leray_6553538	1	Cnidaria	Staurozoa	Stauromedusae		+	-
HE2.Leray_6571642	184	Cnidaria	Staurozoa	Stauromedusae	*Craterolophus convolvulus*	+	-
HE7.Leray_3802459	570	Echinodermata	Asteroidea	Forcipulatida	*Asterias rubens*	+	-
HE3.Leray_388102	85	Echinodermata	Asteroidea	Forcipulatida	*Marthasterias glacialis*	+	-
HF4.Leray_6293728	71	Echinodermata	Echinoidea	Clypeasteroida	*Echinocyamus pusillus*	+	+
HE8.Leray_7326980	315	Echinodermata	Echinoidea	Echinoida	*Psammechinus miliaris*	+	-
HE6.Lobo_7886165	1	Echinodermata	Echinoidea	Spatangoida		+	-
TF3.Leray_6591339	2079	Echinodermata	Echinoidea	Spatangoida	*Brissopsis lyrifera*	+	+
HF7.Leray_1843674	94	Echinodermata	Echinoidea	Spatangoida	*Echinocardium cordatum*	+	-
TS5.Lobo_6025603	11	Echinodermata	Holothuroidea	Dendrochirotida	*Thyone fusus*	+	+
TS3.Leray_6733304	1027065	Echinodermata	Ophiuroidea	Ophiurida		+	+
TS1.Leray_663710	3	Echinodermata	Ophiuroidea	Ophiurida	*Acrocnida brachiata*	-	+
TF1.Lobo_2726978	298	Echinodermata	Ophiuroidea	Ophiurida	*Ophiothrix fragilis*	-	+
TF1.Leray_2426830	16603	Echinodermata	Ophiuroidea	Ophiurida	*Ophiura albida*	+	+
TF5.Leray_2879711	1	Echinodermata	Ophiuroidea	Ophiurida	*Ophiura sarsii*	-	+
HF3.Leray_7012508	44	Gastrotricha	_	Macrodasyida	*Macrodasys* sp.	+	-
HE1.Lobo_948618	14	Gnathostomulida		Bursovaginoidea	*Gnathostomula armata*	+	-
TS2.Leray_4506244	1	Mollusca	Bivalvia	Lucinoida	*Thyasira equalis*	-	+
HF3.Leray_7058438	371	Mollusca	Bivalvia	Myoida	*Corbula gibba*	+	+
HE1.Lobo_894587	22	Mollusca	Bivalvia	Mytiloida	*Mytilus edulis*	+	-
TS1.Lobo_4571224	4	Mollusca	Bivalvia	Nuculida	*Nucula nucleus*	-	+
TS3.Leray_6727248	56213	Mollusca	Bivalvia	Veneroida	*Abra nitida*	+	+
HE4.Lobo_4121128	25	Mollusca	Bivalvia	Veneroida	*Dosinia lupinus*	+	+
TF5.Leray_2915847	1911	Mollusca	Bivalvia	Veneroida	*Kurtiella bidentata*	+	+
TS6.Leray_5683559	2	Mollusca	Bivalvia	Veneroida	*Lucinoma borealis*	-	+
HF1.Leray_2592679	33	Mollusca	Bivalvia	Veneroida	*Spisula subtruncata*	+	-
HE7.Leray_3779267	14392	Mollusca	Bivalvia	Veneroida	*Tellimya ferruginosa*	+	+
HF5.Lobo_3246886	1	Mollusca	Cephalopoda	Sepiida	*Sepietta neglecta*	+	-
TS1.Lobo_4750257	2	Mollusca	Gastropoda	Cephalaspidea		-	+
TS1.Lobo_4792606	2	Mollusca	Gastropoda	Cephalaspidea		-	+
HF8.Lobo_5143779	2	Mollusca	Gastropoda	Littorinimorpha	*Euspira nitida*	+	-
HE3.Lobo_4838288	34	Mollusca	Gastropoda	Neogastropoda	*Mangelia attenuata*	+	+
HF6.Lobo_2622544	37	Mollusca	Gastropoda	Neogastropoda	*Nassarius nitidus*	+	-
HE2.Lobo_1993552	50	Mollusca	Gastropoda	Nudibranchia		+	-
HE6.Leray_2935130	2	Mollusca	Gastropoda	Nudibranchia		+	-
HF1.Leray_2520121	559	Mollusca	Gastropoda	Nudibranchia	*Favorinus branchialis*	+	-
HE2.Lobo_1978270	5	Mollusca	Gastropoda	Nudibranchia	*Onchidoris muricata*	+	-
HE2.Lobo_1939813	155	Mollusca	Gastropoda	Nudibranchia	*Polycera quadrilineata*	+	-
HE2.Lobo_1938412	10	Mollusca	Gastropoda	Nudibranchia	*Polycera quadrilineata*	+	-
HF5.Leray_3991765	847	Mollusca	Gastropoda	Pulmonata	*Microhedyle glandulifera*	+	-
HF4.Leray_6295954	2965	Mollusca	Gastropoda	Sacoglossa	*Elysia viridis*	+	+
HF5.Lobo_3167773	166	Mollusca	Gastropoda	Sorbeoconcha	*Onoba semicostata*	+	-
HE4.Lobo_4138137	2	Mollusca	Gastropoda	Sorbeoconcha	*Pusillina inconspicua*	+	-
TS1.Lobo_4644275	2	Nemertea	Anopla	_	*Cerebratulus* sp.	+	+
HE4.Lobo_4203493	3	Nemertea	Palaeonemertea	_	*Carinina ochracea*	+	-
TF1.Lobo_2662495	1	Nemertea	Palaeonemertea	_	*Hubrechtella dubia*	-	+
HF7.Lobo_5876008	353	Phoronida	_	_	*Phoronis muelleri*	+	-
HE8.Lobo_843910	13	Porifera	Demospongiae	Chondrillida	*Halisarca dujardini*	+	-
HE4.Leray_3148053	1664	Porifera	Demospongiae	Suberitida	*Halichondria panicea*	+	+
TS5.Leray_1547671	2628	Priapulida	Priapulimorpha	Priapulimorphida	*Priapulus caudatus*	+	+
HF5.Leray_3885266	5	Rotifera	Eurotatoria	Flosculariaceae	*Testudinella clypeata*	+	-
HE3.Leray_357208	2	Rotifera	Monogononta	Ploima		+	-
HF8.Lobo_5184437	1	Sipuncula	Sipunculidea	Golfingiida	*Golfingia vulgaris*	+	-
TS1.Lobo_4586276	14	Xenacoelomorpha	_	Acoela	*Archaphanostoma* sp.	-	+
TS3.Lobo_1178177	4	Xenacoelomorpha	_	Acoela	*Childia macroposthium*	-	+
HF9.Lobo_7719366	2	Xenacoelomorpha	_	Acoela	*Haplogonaria viridis*	+	-
HF9.Lobo_7734506	1	Xenacoelomorpha	_	Acoela	*Notocelis Gullmarnensis*	+	-
**18Sa**							
OTU ID	Nb of reads	Phylum	Class	Order	*Species*	HI	GF
TF5.SSU_460284	121639	Annelida	_	_		+	+
TS3.SSU_470635	59	Annelida	_	_		-	+
HF9.SSU_7624	12	Annelida	Clitellata	Enchytraeida	*Grania* sp.	+	-
TF5.SSU_453927	2687	Annelida	Clitellata	Haplotaxida	*Tubificoides insularis*	+	+
HF3.SSU_985477	1090	Annelida	Polychaeta	_	*Aricia* sp.	+	+
HF6.SSU_322303	10	Annelida	Polychaeta	_	*Protodriloides chaetifer*	+	-
HF4.SSU_622170	1	Annelida	Polychaeta	_	*Scalibregma inflatum*	+	-
HF9.SSU_25735	3753	Annelida	Polychaeta	_	*Trilobodrilus heideri*	+	-
TS3.SSU_480632	189	Annelida	Polychaeta	Phyllodocida	*Aphrodita* sp.	-	+
HE6.SSU_371492	49226	Annelida	Polychaeta	Phyllodocida	*Brania* sp.	+	+
HE4.SSU_913344	37252	Annelida	Polychaeta	Phyllodocida	*Glycera* sp.	+	+
HF5.SSU_997904	64	Annelida	Polychaeta	Phyllodocida	*Glycinde armigera*	+	+
TS5.SSU_870099	69	Annelida	Polychaeta	Phyllodocida	*Goniada maculata*	-	+
TF6.SSU_42415	2	Annelida	Polychaeta	Phyllodocida	*Harmothoe imbricata*	-	+
HE6.SSU_350003	5	Annelida	Polychaeta	Phyllodocida	*Myrianida* sp.	+	-
HF6.SSU_324605	2	Annelida	Polychaeta	Phyllodocida	*Nereis pelagica*	+	-
HE7.SSU_239005	67220	Annelida	Polychaeta	Phyllodocida	*Pisione remota*	+	+
HE2.SSU_637269	49	Annelida	Polychaeta	Phyllodocida	*Platynereis dumerilii*	+	-
HE8.SSU_832291	1	Annelida	Polychaeta	Phyllodocida	*Progoniada regularis*	+	-
HE8.SSU_834197	1	Annelida	Polychaeta	Sabellida	*Fabriciola liguronis*	+	-
HF2.SSU_202737	4	Annelida	Polychaeta	Sabellida	*Laeospira corallinae*	+	-
HE2.SSU_640060	3	Annelida	Polychaeta	Sabellida	*Myriochele* sp.	+	-
TS5.SSU_869292	123	Annelida	Polychaeta	Spionida	*Apistobranchus* sp.	-	+
TS3.SSU_517096	1407	Annelida	Polychaeta	Spionida	*Laonice* sp.	-	+
HE3.SSU_123438	1952	Annelida	Polychaeta	Spionida	*Spio* sp.	+	+
TS5.SSU_882766	60	Annelida	Polychaeta	Terebellida	*Diplocirrus glaucus*	-	+
HF2.SSU_193854	1	Annelida	Polychaeta	Terebellida	*Flabelligera* sp.	+	-
TF6.SSU_63146	669	Annelida	Polychaeta	Terebellida	*Pectinaria* sp.	-	+
TS5.SSU_883475	4155	Annelida	Polychaeta	Terebellida	*Terebellides stroemii*	-	+
TF4.SSU_139713	193	Arthropoda	Branchiopoda	_		-	+
HE5.SSU_184679	149	Arthropoda	Malacostraca	_		+	-
HE8.SSU_832214	1	Arthropoda	Malacostraca	Decapoda	*Nikoides* sp.	+	-
HF5.SSU_994971	7	Arthropoda	Malacostraca	Decapoda	*Praebebalia longidactyla*	+	-
TF6.SSU_56595	65992	Arthropoda	Maxillopoda	_		+	+
HF9.SSU_15855	31800	Arthropoda	Maxillopoda	_		+	+
HF2.SSU_208480	21241	Arthropoda	Maxillopoda	_		+	+
TS2.SSU_812824	433	Arthropoda	Maxillopoda	_		+	+
TF3.SSU_955499	185	Arthropoda	Maxillopoda	_		+	+
TF5.SSU_470101	360	Arthropoda	Maxillopoda	Harpacticoida	*Typhlamphiascus typhlops*	-	+
HE1.SSU_864375	1160	Arthropoda	Ostracoda	Podocopida	*Hemicytherura kajiyamai*	+	+
HE7.SSU_253407	2584	Arthropoda	Ostracoda	Podocopida	*Loxocorniculum mutsuense*	+	+
HE5.SSU_181011	1	Arthropoda	Pycnogonida	Pantopoda	*Anoplodactylus californicus*	+	-
HE2.SSU_646490	123	Arthropoda	Pycnogonida	Pantopoda	*Callipallene* sp.	+	-
HE2.SSU_638224	23	Bryozoa	_	_		+	-
HE6.SSU_373369	2	Bryozoa	Stenolaemata	Cyclostomatida	*Plagioecia patina*	+	-
HE1.SSU_850917	4	Bryozoa	Stenolaemata	Cyclostomatida	*Tubulipora lobifera*	+	-
TF5.SSU_412099	18	Cephalorhyncha	Kinorhyncha	Homalorhagida	*Pycnophyes kielensis*	-	+
HE7.SSU_239963	45	Chordata	Actinopteri	Perciformes	*Hypseleotris* sp.	+	+
HE3.SSU_123107	4	Chordata	Ascidiacea	_		+	-
HF9.SSU_12142	727	Chordata	Ascidiacea	Phlebobranchia	*Ascidiella* sp.	+	+
HF4.SSU_611685	114	Chordata	Ascidiacea	Phlebobranchia	*Corella inflata*	+	+
HE2.SSU_639404	209	Chordata	Ascidiacea	Stolidobranchia	*Molgula* sp.	+	-
HE9.SSU_314754	616	Chordata	Ascidiacea	Stolidobranchia	*Styela plicata*	+	-
HE8.SSU_834024	11058	Chordata	Leptocardii	_	*Branchiostoma* sp.	+	-
TF1.SSU_674740	2212	Cnidaria	Anthozoa	Actiniaria	*Nematostella vectensis*	+	+
TS3.SSU_472524	2741	Cnidaria	Hydrozoa	_		+	+
TS3.SSU_518760	7860	Cnidaria	Hydrozoa	Anthoathecata	*Euphysa* sp.	+	+
HE2.SSU_639670	1	Cnidaria	Hydrozoa	Leptothecatha	*Abietinaria filicula*	+	-
TF4.SSU_152912	61418	Echinodermata	_	_		+	+
HE5.SSU_186025	8038	Echinodermata	_	_		+	+
TF4.SSU_155631	5491	Echinodermata	_	_		+	+
TS5.SSU_881395	25	Echinodermata	_	_		-	+
HE4.SSU_914821	1	Echinodermata	Holothuroidea	Apodida	*Leptosynapta* sp.	+	-
HF9.SSU_2577	1006	Gastrotricha	_	Chaetonotida	*Chaetonotus* sp.	+	+
HE7.SSU_244283	249	Gastrotricha	_	Macrodasyida	*Diplodasys meloriae*	+	-
HF5.SSU_996540	161	Gastrotricha	_	Macrodasyida	*Lepidodasys* sp.	+	-
HF5.SSU_995416	636	Gastrotricha	_	Macrodasyida	*Macrodasys* sp.	+	-
HF2.SSU_192734	479	Gastrotricha	_	Macrodasyida	*Macrodasys* sp.	+	-
HF7.SSU_385728	6934	Gastrotricha	_	Macrodasyida	*Mesodasys* sp.	+	+
HE7.SSU_242889	3013	Gastrotricha	_	Macrodasyida	*Tetranchyroderma thysanophorum*	+	-
HF1.SSU_770513	339	Gastrotricha	_	Macrodasyida	*Thaumastoderma ramuliferum*	+	-
HF1.SSU_760431	5	Gastrotricha	_	Macrodasyida	*Urodasys* sp.	+	-
TF6.SSU_44832	3816	Mollusca	Bivalvia	_		+	+
HF2.SSU_208561	14	Mollusca	Bivalvia	Anomalodesmata		+	+
HF8.SSU_788507	1	Mollusca	Bivalvia	Limoida	*Limaria hians*	+	-
TF3.SSU_924397	11725	Mollusca	Bivalvia	Veneroida	*Abra* sp.	+	+
HE9.SSU_317977	1982	Mollusca	Bivalvia	Verenoida	*Arctica islandica*	+	+
TF4.SSU_132537	1581	Mollusca	Gastropoda	Neogastropoda	*Nassarius festivus*	+	+
HF1.SSU_779114	65	Nematoda	Chromadorea	Araeolaimida	*Odontophora* sp.	+	+
TF6.SSU_48167	2940	Nematoda	Chromadorea	Araeolaimida	*Sabatieria* sp.	+	+
TF1.SSU_710679	639	Nematoda	Chromadorea	Chromadorida		+	+
HF2.SSU_192072	2	Nematoda	Chromadorea	Chromadorida	*Chromadora nudicapitata*	+	-
HF1.SSU_759758	4	Nematoda	Chromadorea	Plectida		+	-
HF9.SSU_20251	636	Nematoda	Desmodorida	Microlaimidae		+	+
HE3.SSU_124287	13	Nematoda	Enoplea	Enoplida	*Enoploides* sp.	+	-
HE3.SSU_110275	8	Nematoda	Enoplea	Enoplida	*Enoplus* sp.	+	-
HE5.SSU_188855	27	Nematoda	Enoplea	Enoplida	*Symplocostoma* sp.	+	+
TS6.SSU_587229	493	Nematoda	Enoplea	Enoplida	*Viscosia viscosa*	+	+
TF3.SSU_938615	642	Nemertea	_	_		+	+
TF6.SSU_49192	265	Nemertea	Anopla	_	*Cerebratulus marginatus*	+	+
HE4.SSU_908113	877	Nemertea	Anopla	_	*Lineus bilineatus*	+	+
HF9.SSU_3582	6	Nemertea	Paleonemertea	_	*Callinera grandis*	+	-
HE3.SSU_121696	12053	Nemertea	Paleonemertea	_	*Cephalothrix filiformis*	+	+
TF5.SSU_434928	1760	Nemertea	Paleonemertea	_	*Hubrechtella dubia*	+	+
TS2.SSU_818002	1	Platyhelminthes	Rhabditophora	Cestoda		-	+
HE9.SSU_303121	1939	Platyhelminthes	Rhabditophora	Haplopharyngida	*Haplopharynx rostratus*	+	-
HF1.SSU_773830	1	Platyhelminthes	Rhabditophora	Prolecithophora	*Allostoma neostiliferum*	+	-
HE2.SSU_650311	8	Platyhelminthes	Rhabditophora	Prolecithophora	*Cylindrostoma* sp.	+	-
HE5.SSU_177399	4	Platyhelminthes	Rhabditophora	Prolecithophora	*Euxinia baltica*	+	-
HF9.SSU_23023	8367	Platyhelminthes	Rhabditophora	Prolecithophora	*Plagiostomum cinctum*	+	+
TS2.SSU_822141	938	Platyhelminthes	Rhabditophora	Prolecithophora	*Plagiostomum cuticulata*	-	+
TF6.SSU_52738	214	Platyhelminthes	Rhabditophora	Prolecithophora	*Plagiostomum striatum*	-	+
TF5.SSU_433159	2	Platyhelminthes	Rhabditophora	Prolecithophora	*Ulianinia mollissima*	-	+
HF9.SSU_24513	59	Platyhelminthes	Rhabditophora	Proseriata	*Monocelis lineata*	+	+
HF2.SSU_201740	2	Platyhelminthes	Rhabditophora	Rhabdocoela	*Phonorhynchus helgolandicus*	+	-
TS6.SSU_592673	245	Platyhelminthes	Rhabditophora	Rhabdocoela	*Proxenetes* sp.	+	+
HF4.SSU_616041	771	Platyhelminthes	Rhabditophora	Seriata		+	-
HE3.SSU_117223	181	Porifera	Calcarea	_		+	+
HE7.SSU_223989	12	Porifera	Demospongiae	Chondrillida	*Halisarca dujardini*	+	-
HF9.SSU_26977	8	Porifera	Demospongiae	Clionaida	*Spheciospongia vesparium*	+	-
HE6.SSU_383060	3	Sipuncula	Sipunculidea	Golfingiida	*Phascolopsis gouldii*	+	-
HE6.SSU_348954	2	Tardigrada	Eutardigrada	Parachela	*Halobiotus crispae*	+	-
TF3.SSU_927927	2	Xenacoelomorpha	_	_		-	+
HE3.SSU_116025	28	Xenacoelomorpha	_	Acoela	*Archaphanostoma* sp.	+	+
HF9.SSU_26335	1	Xenacoelomorpha	_	Acoela	*Archaphanostoma* sp.	+	-
TS2.SSU_815721	2	Xenacoelomorpha	_	Acoela	*Childia* sp.	-	+
TS2.SSU_815970	1	Xenacoelomorpha	_	Acoela	*Childia* sp.	-	+
HF2.SSU_190395	2386	Xenacoelomorpha	_	Acoela	*Eumecynostomum* sp.	+	-
HF1.SSU_758202	74	Xenacoelomorpha	_	Acoela	*Haplogonaria* sp.	+	+
HF9.SSU_13290	5	Xenacoelomorpha	_	Nemertodermatida	*Flagellophora apelti*	+	-
TS6.SSU_601153	28	Xenacoelomorpha	_	Nemertodermatida	*Nemertoderma westbladi*	-	+

**Table 9.** Invasive species (in bold) and species on alert lists (not bold) found in the samples. X indicates where the species were found.****

**Species**	**Phylum**	**COI**		**18S**	
		Hållö island	Gullmarn Fjord	Hållö island	Gullmarn Fjord
***Acartia tonsa***	Arthropoda	x	x		
***Alexandrium ostenfeldii***	Dinoflagellata			x	x
*Bonnemaisonia hamifera*	Rhodophyta	x	x	x	
*Penilia avirostris*	Arthropoda	x	x		
*Thalassiosira punctigera*	Bacillariophyta	x			

**Table 10.** Taxonomic composition and relative abundance (% of the total number of specimens) of Xenacoelomorpha species in Gullmarn Fjord and Hållö sites.

		**Gullmarn Fjord**		**Hållö**	
	**Taxon**	**Siphoning**	**Flotation with fresh water**	**Flotation with MgCl2 solution**	**Flotation with fresh water**
	** Acoela **				
1	*Haploposthia rubropunctata *	1.03	0	0	0
2	*Childia brachyposthium *	3.78	0	0	0
3	*Childia submaculatum*	1.03	0	0	0
4	*Childia trianguliferum*	2.06	0	0	0
5	*Childia crassum*	3.44	0	0	0
6	*Childia* sp.	25.09	0	0	0
7	*Mecynostomum tenuissimum*	43.99	0	0	0
8	*Mecynostomum auritum*	0.34	0	0	0
9	cf. *Eumecynostomum altitudi*	4.81	0	0	0
10	*Philactinoposthia* sp.	0.34	0	0	0
11	Acoela sp.	2.06	100	88.71	0
12	*Faerlea glomerata*	3.09	0		
13	*Archaphanostoma* sp.	0.34	0	0.81	0
14	*Postmecynostomum glandulosum*	0	0	2.42	0
15	*Paramecynostomum* sp.	0	0	0.81	0
16	*Eumecynostomum macrobursalium*	0	0	0.81	0
17	*Isodiametra* sp.	0	0	0.81	0
18	*Haplogonaria viridis/Archocelis macrorhabditis*	0	0	5.65	0
	** Nemertodermatida **				
19	*Nemertoderma westbladi*	8.25	0	0	0
20	*Flagellophora apelti*	0.34	0	0	0

**Table 11.** Total number of Xenacoelomorpha taxa or OTUs distinguished based on morphology (Table 10), 18S and COI from different sampling sites and extraction methods (placement of OTUs is based on 80% similarity threshold, Suppl. materials 2, 3)

**Site / extraction method**	**morphology-based**	**18S**	**COI (Lobo)**	**COI (Leray)**
Hållo, flotation with MgCl2	7	11	8	6
Hållö, flotation with fresh water	0	15	11	6
Hållö, total	7	16	12	7
Gullmarn Fjord, siphoning	15	11	9	4
Gullmarn Fjord, flotation with fresh water	1	13	2	0
Gullmarn Fjord, total	15	19	10	4

**Table 12.** Total number of nematode taxa or OTUs distinguished based on morphology (after Holovachov et al. 2017), 18S and COI from different sampling sites and extraction methods (placement of OTUs is based on 80% similarity threshold, Suppl. materials 2, 3)

**Site / extraction method**	**morphology-based**	**18S**	**COI (Lobo)**	**COI (Leray)**
Hållo, flotation with MgCl2	88	71	12	11
Hållö, flotation with fresh water	101	78	14	14
Hållö, total	107	95	16	17
Gullmarn Fjord, siphoning	81	47	8	4
Gullmarn Fjord, flotation with fresh water	102	67	4	2
Gullmarn Fjord, total	113	78	9	4
